# Successful containment of a nosocomial outbreak of Burkholderia cepacia in a special care baby unit of a base hospital in Sri Lanka

**DOI:** 10.1186/2047-2994-4-S1-P264

**Published:** 2015-06-16

**Authors:** YC Waniganayake, UA Wijetunga, L Karunanayake, R Abeysinghe

**Affiliations:** 1 Base Hospital Avissawella, Avissawella, Sri Lanka; 2 Department of Bacteriology, Medical Research Institute, Colombo, Sri Lanka

## Introduction

*Burkholderia cepacia* a known cause of nosocomial infection, is capable of surviving in nutrient-poor water. It’s intrinsically resistant to most antibiotics.

## Objectives

To present the first nosocomial outbreak of *B. cepacia* reported in Sri Lanka.

## Methods

Patient and environmental sampling data obtained during the outbreak were retrospectively reviewed. Blood cultures(BCs) were performed using the BacT/ALERT® automated system. Environmental samples were cultured in brain heart infusion broths. API 20NE was used for biochemical identification. Antibiograms(ABs) were carried out according to CLSI guidelines.

## Results

Over 6 days in May 2013, 4 BCs out of 14 in the special care baby unit of Base Hospital Avissawella, grew *B. cepacia*. BCs became positive after a mean of 3.5 days since admission. All four neonates developed high levels of inflammatory markers and thrombocytopaenia. Organism showed identical ABs in all isolates, with sensitivity to meropenem, ceftazidime, ciprofloxacin and resistance to trimethoprim and aminoglycosides. All babies were successfully treated with meropenem.

A 10% dextrose solution used for the neonates grew *B. Cepacia* on culture. This solution is not readily available in our setting and it’s prepared in the unit, using 5% and 50% dextrose combination. It was used for different babies at multiple times throughout the day by several nurses. Other samples that grew *B. cepacia* included a bottle of normal saline used as a multiple dose vial for preparation of intravenous (IV) drugs, a gallipot of sterile water used for suction of airway of a ventilated baby, 2 incubator humidifiers and a ventilator humidifier. ABs were identical to those of patient isolates. Unit policy was changed to preparing IV fluids for all babies at the same time, twice a day by a designated nurse wearing sterile gloves. Remaining IV fluid was discarded. Single use normal saline bottles were purchased. A fresh gallipot of sterile water was used for airway suctioning each time. Humidifier cleaning technique was optimized.

## Conclusion

In a resource limited tropical setting, cessation of the use of multiple dose IV fluid vials and simple hygienic measures proved to be effective in controlling and preventing outbreaks of *B. cepacia*.

## Disclosure of interest

None declared.

